# A novel screening method of DNA methylation biomarkers helps to improve the detection of colorectal cancer and precancerous lesions

**DOI:** 10.1002/cam4.6511

**Published:** 2023-10-25

**Authors:** Yuan Li, Bin Li, Rou Jiang, Leen Liao, Chunting Zheng, Jie Yuan, Liuhong Zeng, Kunling Hu, Yuyu Zhang, Weijian Mei, Zhigang Hong, Binyi Xiao, Lingheng Kong, Kai Han, Jinghua Tang, Wu Jiang, Zhizhong Pan, Shenyan Zhang, Peirong Ding

**Affiliations:** ^1^ Department of Colorectal Surgery Sun Yat‐sen University Cancer Center, State Key Laboratory of Oncology in South China, Collaborative Innovation Center of Cancer Medicine Guangzhou China; ^2^ Beijing BGI‐GBI Biotech Co., Ltd Beijing China; ^3^ Department of Cancer Prevention Center Sun Yat‐sen University Cancer Center Guangzhou China; ^4^ Department of General Surgery The Fifth Affiliated Hospital of Southern Medical University Guangzhou China; ^5^ BGI Genomics, BGI‐Shenzhen Shenzhen China

**Keywords:** cell‐free DNA, colorectal cancer, DNA methylation biomarkers, noninvasive screening

## Abstract

**Background:**

Colorectal cancer (CRC) is one of the most common malignancies, and early detection plays a crucial role in enhancing curative outcomes. While colonoscopy is considered the gold standard for CRC diagnosis, noninvasive screening methods of DNA methylation biomarkers can improve the early detection of CRC and precancerous lesions.

**Methods:**

Bioinformatics and machine learning methods were used to evaluate CRC‐related genes within the TCGA database. By identifying the overlapped genes, potential biomarkers were selected for further validation. Methylation‐specific PCR (MSP) was utilized to identify the associated genes as biomarkers. Subsequently, a real‐time PCR assay for detecting the presence of neoplasia or cancer of the colon or rectum was established. This screening approach involved the recruitment of 978 participants from five cohorts.

**Results:**

The genes with the highest specificity and sensitivity were Septin9, AXL4, and SDC2. A total of 940 participants were involved in the establishment of the final PCR system and the subsequent performance evaluation test. A multiplex TaqMan real‐time PCR system has been illustrated to greatly enhance the ability to detect precancerous lesions and achieved an accuracy of 87.8% (95% CI 82.9–91.5), a sensitivity of 82.7% (95% CI 71.8–90.1), and a specificity of 90.1% (95% CI 84.3–93.9). Moreover, the detection rate of precancerous lesions of this assay reached 55.0% (95% CI 38.7–70.4).

**Conclusion:**

The combined detection of the methylation status of SEPT9, SDC2, and ALX4 in plasma holds the potential to further enhance the sensitivity of CRC detection.

## 1 INTRODUCTION

Colorectal cancer (CRC) is one of the most common cancers in the world.^1^, ^2^, ^3^, ^4^ In China, more than 376,000 new cases occur per year, ranking third in malignancy.^5^ Due to the extended evolutionary period from polyps to cancer, effective CRC screening holds significant importance. Actively promoting clinical screening for CRC can effectively reduce morbidity, mortality, and enhance patient survival.^6^ Developed countries have witnessed a decline in CRC mortality in recent years, attributed to early detection and treatment for both early‐stage colorectal cancer and precancerous lesions through screening.^7^, ^8^ Currently, the most commonly used CRC screening methods are colonoscopy and fecal occult blood test/fecal immunochemical detection, which are relatively effective. Early screening methods can significantly reduce the mortality of CRC.^9^ However, the utilization rate is not high, mainly because the cost of colonoscopy and intestinal preparation is not well complied with by the patients, while fecal occult blood test has many interference factors and low specificity.

Studies have shown that DNA methylation is an early event in the development of CRC.^10^, ^11^ Thus, specific gene methylation might be used as a molecular marker for early tumor screening.^12^, ^13^, ^14^, ^15^ The corresponding samples are feces, serum, or plasma, which are less invasive and have higher compliance, and can improve the relatively low screening rate. Among the studied DNA methylation analyses, the SEPT9 gene has received considerable attention due to its efficacy in distinguishing between normal and CRC samples.^16^ Abnormal SEPT9 methylation has been significantly detected in colorectal cancer compared to normal colorectal tissue. SEPT9 gene methylation is a specific marker in the early development of colorectal cancer, which follows the shedding of cancer cells into the blood. Detection of methylation in the gamma region of the SEPT9 v2 transcriptome promoter in plasma has enabled early screening for colorectal cancer.^17^

There are numerous methods currently used to detect DNA methylation, the most common being methylation‐specific PCR (MSP). Single‐gene methylation has sensitivity limitations in detecting CRC, especially in detecting advanced adenomas.^18^, ^19^ Despite the second‐generation SEPT9 methylation detection kit improved its sensitivity to 27.4%, it still fails to meet clinical requirements.^20^, ^21^ Notably, an analysis of methylated SEPT9 in Korean human species found that only 39.6% of colorectal cancer subjects showed positive methylation of the SEPT9 gene, and no methylation of the SEPT9 gene was detected in adenomas.^22^ This might relate to the fact that though SEPT9 gene methylation is widespread in colorectal progressive adenomas, there is a lag in its release into the bloodstream after it invades the blood vessels in advanced CRC.^16^ A single SEPT9 gene methylation assay may lead to serious missed and misidentified tests. As a result, some scholars have used polygenic methylation combination assays to obtain better results.^23^

Cumulative studies over the past decades have included the P16, ALX4, SEPT9, and TMEFF2 genes as candidate genes for early screening of CRC,^15^, ^18^, ^24^, ^25^, ^26^ and a growing number of DNA methylation candidate genes are being unearthed.

SDC2 is involved in cell proliferation, migration, and cell‐substrate interactions.^27^ DNA microarray analysis of CRC samples revealed a high methylation rate of approximately 95% for the SDC2 gene, regardless of the cancer stage.^28^ SDC2 gene has a promoter methylation rate of 89.4% in cell‐free DNA of CRC patients and 81.1% in adenoma stage cancer patients.^29^ Another study found that SDC2 was 100% methylated in premature tumor tissue, 90.6% methylated in adenomatous stage tissue, and 90.9% methylated in proliferative polyp tissue, while no methylation was detected in normal tissue. In stool DNA tests for SDC2 methylation, the overall sensitivity for detecting CRC was 90.0%, and for detecting small polyps, it was 33.3%, with a specificity of 90.9%.^30^ Serologic tests also demonstrated a sensitivity of 87.0% and a specificity of 95.2% for SDC2 methylation in clinical screening of colorectal cancer. Importantly, the sensitivity at stage I was 92.3%, indicating the potential of SDC2 methylation as a blood‐based DNA test for early detection of CRC.^28^

ALX4 encodes a paired‐like homeodomain transcription factor expressed in the mesenchyme of developing bones, limbs, hair, teeth, and mammary tissue. Promoter methylation detection of ALX4 has shown a sensitivity and specificity of 68% and 88% in CRC distinguish, respectively, and can be used as a molecular marker for early screening of colorectal cancer.^31^ Furthermore, a combined assay using methylated SEPT9 and ALX4 detection has demonstrated an improvement in the detection of precancerous lesions compared to SEPT9 alone, increasing from 29% to 37%.^19^

Up to date, the application of polygenic methylation combination assays to screening of CRC has not been well studied. Therefore, our research aims to evaluate the application value of detecting the methylation status of SEPT9, SDC2, and ALX4 genes in cell‐free DNA from CRC patients, precancerous lesion patients, and healthy individuals. We will use colonoscopy and pathology test results as the “gold standard” to assess the degree of MSP and its potential in colorectal cancer screening.

## 2 MATERIALS AND METHODS

### 2.1 Biomarkers selection

Clinical characteristics and molecular profiling, including level three data of Illumina Infinium human DNA methylation 450 K array for a cohort of 396 colorectal adenocarcinoma samples and 35 matched adjacent normal tissue samples, as well as a cohort of 297 colon adenocarcinoma samples and 38 matched adjacent normal tissue samples; and level three data of Illumina HiSeq2000 RNA Sequencing for a cohort of 632 colorectal adenocarcinoma samples and 45 matched adjacent normal tissue samples, as well as a cohort of 459 colon adenocarcinoma samples and 41 matched adjacent normal tissue samples, were obtained from The Cancer Genome Atlas (TCGA) and downloaded from the Broad GDAC Firehose (http://gdac.broadinstitute.org/). We used the absolute value of delta‐beta with a cutoff of 0.2, the beta value of control samples with a cutoff of 0.1, the student's *t‐*test method with a 95% confidence interval, and the Benjamin–Hochberg procedure^32^ to control the false discovery rate (FDR) at a significance level of 0.01 to identify the differentially methylated position (DMP).^33^ The differentially expressed gene (DEG) was determined by the student's *t*‐test method with a 95% confidence interval and the Benjamin–Hochberg procedure to control the false discovery rate (FDR) at a significance level of 0.01 from the logarithm of RSEM normalized expression value to the base 2. The correlation coefficient of Spearman, Pearson, and Kendall was calculated between the methylation data and the mRNA sequencing data.^34^ Genes with DMP, DEG, and negative Spearman correlation coefficient and have been reported in the literature were selected as candidate biomarkers. The least absolute shrinkage and selection operator (LASSO) regression algorithm was used to select biomarkers from DMP and a weight scoring system was established by randomly repeating a thousand times with LASSO to get the best sensitivity and specificity in 70% data as training cohort and 30% data as testing cohort.

### 2.2 Biomarkers confirmation

On the basis of the database and the literature analysis,^35^, ^36^, ^37^, ^38^, ^39^, ^40^ a total of 56 genes were selected as the most likely potential methylation‐specific biomarkers for CRC early screening, of which ALX4 and CHFR were obtained using Illumina Infinium 27K arrays directly.^41^ From them, we chosen 22 genes according to weight scores for clinical sample detection to find out the accurate biomarkers (Appendix S1).

### 2.3 Primer designation and selection

For each of the 22 candidate genes, we designed one to seven pairs of MSP and the unmethylation‐specific primers (USP) of the same sites using MethPrimer online software simultaneously.^42^ In total, 74 pairs of MSP and USP primers for all 22 candidate genes were gained. The MSP and corresponding USP primers were in the CpG‐rich regions of the promoters or CpG islands of these genes. Methylated DNA (Millipore) was used as a positive reference for gene methylation study, while genomic DNA (BioChain) was used as a negative reference. β‐actin (ACTB) was used as an internal control to determine the validity of the result.^18^ Primers were ordered from invitrogen, and the redundant were filtered out as described: The ability for template binding of the 74 pairs of primers was tested using positive and negative references and selected when the pair of MSP and USP primers amplified the correct products with right sequences sequenced by 3730 sequencers. Thus, 22 pairs of MSP primers covering 17 genes were obtained and used to amplify seven CRC genomic DNA extracted from CRC tissues and the corresponding genomic DNA extracted from the seven CRC patients' peripheral blood cells. MSP primers that amplified correctly for the former and meanwhile blankly amplified for the latter were opted as the usable biomarkers and ranged by tissue sensitivity from high to inferior. Totally, eight pairs of MSP primers covered eight genes that were supposed to meet our expectations. These genes were SEPT9, ALX4, RASSF2, SFRP2, SDC2, TFPI2, NDRG4, and CHFR. Finally, cell‐free DNA (cf‐DNA) extracted from 80 asymptomatic healthy donors' plasma was checked by each of the eight pairs of MSP primers through methylation‐specific PCR unless that was insufficient. The specificity of these opted biomarkers for plasma ranged from 82.5% to 98.3% (Appendix S1). As a result, the top three genes SEPT9, ALX4, and SDC2 were chosen as the final biomarkers for their best tissue sensitivity and plasma specificity.

### 2.4 Probe designation and selection

We designed two to four TaqMan probes for each of the three amplicons and ACTB abovementioned with the online tool IDT, available at https://sg.idt‐dna.com/Scitools/Applications/RealTimePCR/. TaqMan real‐time PCR was performed on an ABI 7500 real‐time PCR system (Thermo) to test each probe of 4 genes. The most appropriate probe which may give out a strong and stable fluorescent signal and a distinction between positive samples and negative samples according to the amplification curves and results was selected. In this way, a multiplex TaqMan real‐time PCR was established.

### 2.5 Participants

Ethics committees of hospitals and BGI approved this program, and informed consent was signed as required. Patients with all stages of CRC were verified by colonoscopy. To determine the disease status and stage of colorectal cancer patients, clinical records were acquired. Healthy individuals were confirmed by multitarget stool DNA test (BGI), and subjects with positive results were required to undergo colonoscopy. Only those with both negative results were recruited as no evidence of disease (NED) at one of the BGI clinical centers. Subjects were at least 20 years old, and the majority were 40 and older. Participants without an history of HIV or herpes virus B or C infection, cancer other than basal cell skin cancer, or symptoms of severe acute or exacerbated chronic disease were required. Additionally, patients who had a history of cancer of the upper respiratory tract and digestive tract and performed any bowel resection other than sigmoid diverticulum resection or interventional clinical study within the previous 30 days were excluded.

### 2.6 Clinical procedure

Basic information of the study and informed consent were solicited from the enrolled eligible subjects at least for the past 1 day before the initiation of colonoscopy preparation. At least 10 mL of peripheral blood from subjects was collected in the EDTA anticoagulant tube (CHGD), mixed, and labeled. Plasma was isolated from whole blood within 4 h since collection by centrifugation (1600 *g*, 10 min, 4°C). The supernatant was recentrifuged (16,000 *g*, 10 min, 4°C) and aliquoted into a coded 2 mL tube (Axygen) and then stored at −80°C in a local participating center before being periodically shipped to BGI. Dry ice was used in transportation to ensure aliquots were maintained at −20°C. Colonoscopy procedures, including biopsy, were performed in each participating center by certified endoscopy physicians following guidelines.^43^ Cancer diagnosis was confirmed by histopathology, staging of biopsy, and surgical specimens, which were performed using routine procedures in each center. Colonoscopy and pathology reports for CRCs, as well as multitarget stool DNA tests for healthy individuals, were abstracted into study forms and e‐mailed to BGI afterward.

### 2.7 Plasma simulation

A simulated plasma solution was prepared by adding a known amount of positive or negative reference that had been broken into ~200 bp with ultrasound to the Custom Bulk Processed Plasma (SeraCare). It was then used as a quality control to monitor the entire experimental process from DNA extraction to data statistics.

### 2.8 DNA purification

Kits from different manufacturers, including TIANGEN, MAGEN, and QIAGEN, were tested following each kit's manual by extracting DNA from the simulated plasma solution. Taking the convenience of delivery, price, and extraction result into account, MagPure Circulating DNA Maxi Kit (Magen) was selected. As is required, cell‐free DNA was captured on magnetic beads from an aliquot that contains ~2 mL of subject plasma, purified, and eluted in a final volume of 55 μL. Concentrations were measured by Qubit 3.0 soon later. The remaining ∼2 mL of plasma was frozen at −80°C and stored in case of need for re‐detection.

### 2.9 DNA conversion

EZ DNA Methylation‐Gold Kit (ZYMO) was used to convert the unmethylated cytosine in the purified DNA to uracil and then captured again with the column, purified through washing steps and eluted in a final volume of 15 μL.

### 2.10 PCR procedure optimization

In order to get the best amplification performance, enzymes from different manufacturers, several amplification systems, and procedures were tested on 7500 devices using positive and negative references as templates. The linear correlation between amplification results and template concentration, coefficient of variance (CV) between replicates, as well as amplification efficiency were considered as criteria in amplification performance. For the purpose of coordinating the different annealing temperatures between primer pairs, a touchdown PCR (TD‐PCR) prior to a multiplex TaqMan qPCR procedure was chosen in view of its excellent performance, of which the HotStart HiTaq DNA Polymerase (Fapon) or High‐Affinity HotStart Taq (TIANGEN) was used and 5 μL of template demanded. Clinical samples test showed a much higher detection rate of colorectal precancerous lesions by using HotStart HiTaq DNA Polymerase (A1, assay1) than that of similar commercially available products. However, a specificity of nearly 50% was its fatal deficiency. The problem was thought to be due to the nonspecific fluorescence generation during the PCR procedure for the detection of fluorescent signals in blank or negative controls occasionally, so the results of the test are not stable or reliable. Thus, we further optimized our experimental system based on the previous experience by changing the polymerase into High‐Affinity HotStart Taq (A2, assay2), which could greatly elevate the specificity after modifying the resulting algorithm.

In short, after optimization of various factors affecting the PCR process and clinical sample testing, the selected option as follows: 20 μL of a mixture of PCR buffer, oligonucleotides, and polymerase were prepared and added to a 96‐well PCR plate, and then 5 μL of bisulfited DNA was added per RXN and amplified with a special PCR procedure on a 7500 device as mentioned above. Two parallel reactions for each subject sample were executed. ACTB was used as an internal control while the positive and negative references were used as the external control to determine the validity of the result. The methylated SEPT9, ALX4, and SDC2 (mSEPT9, mALX4, and mSDC2) were set as the final biomarkers for assessing the risk of CRC. Technical details, such as mixture preparation, oligonucleotide sequences and concentrations, and the amplification parameters setting can be accessed in Appendix S1.

### 2.11 Pathology classification

Participants were assorted into three categories: CRC, precancerous lesions (PL), and no evidence of disease (NED) given the survey of the clinical information and histopathological characteristics. In addition, CRC was subclassified into stages I–IV, and PL was subclassified into advanced adenoma (AA) and non‐advanced adenoma (NA). AA included high‐grade dysplasia (HGD), villous architecture, or large polyps (≥10 mm). Other adenomas were classed as NA.

### 2.12 Statistical analysis

The target population was residents over 40 years old in mainland China or volunteers who were willing to participate in the study. A preliminary analysis of the required sample size was conducted using PASS software (www.ncss.com). The estimated sample size was determined based on an expected sensitivity of 83.0% and specificity of 90.0%. According to a pilot study (Data not shown), the desired width of the confidence interval (CI) was set to be no more than 0.08 for sensitivity and 0.16 for specificity, aiming to achieve a 95% confidence level (*α* = 0.05). Since the patients and NEDs used an equal sample size, the two groups of subjects took the aforementioned maximum value, 362 patients with CRC or PL and 362 NED demanded. Actually, a valid sample pool of 212 CRC, 136 PL, and 369 NED was adopted for A1 and A2 assays.

The diagnostic performance of each gene was assessed by using a univariate unconditional logistic regression algorithm with “glm” R function or receiver operating characteristic curves (ROC) and the areas under the receiver operating characteristic curves (AUCs). Repeated test results in one sample were considered as multiple test results. Single‐factor nonconditioned logistic regression algorithm was assessed with 70% samples as training sets and 30% samples as test sets in repeat 100 times. The cutoff values were defined as the maximal sum of sensitivity and specificity in each gene. Then, the combination performance of each gene was assessed by ROC and AUCs, and determined that if any one of the three genes is positive (that is, the CT value of the gene is smaller than the cutoff of the gene), then the test is positive. The diagnostic performance of the multiplex TaqMan real‐time PCR assay with repeat tests was assessed by balancing algorithm, one‐half algorithm, and two‐thirds algorithm. Balancing algorithm was defined as the sample test result being positive if any of the following conditions are satisfied: (i) the CT of ACTB gene should be ≤40 or else the test result is invalid and needs a retest; (ii) test results are positive for each well (that is, any one of the three genes is positive in a single test with a cutoff of 45, 38, and 40 in mSEPT9, mALX4, and mSDC2 genes, respectively); (iii) the CT value of mALX4 gene is smaller than 38 in a single test; (iv) the CT values for any two genes are less than 40, and the corresponding delta CT values are less than 10. One‐half algorithm was defined as the sample test result being positive if the test result of any good test is positive (any one of the three genes is positive in a single test with a cutoff of 45, 38, and 43 in mSEPT9, mALX4, and mSDC2 genes, respectively, and the corresponding delta CT values are less than 15). The two‐thirds algorithm was defined as the sample test result being positive if test results are positive for each well (that is, any one of the three genes is positive in a single test with a cutoff of 40.67, 36.8, and 36.23 in mSEPT9, mALX4, and mSDC2 genes, respectively). All the statistical analyses were performed using R language 3.5.1 (https://www.r‐project.org) R Core Team, 2018.^44^ SPSS 22.0 (IBM), and online tool http://vassarstats.net/index.html for calculating the 95% confidence intervals.

## 3 RESULTS

### 3.1 Sample enrollment and distribution

**FIGURE 1:**
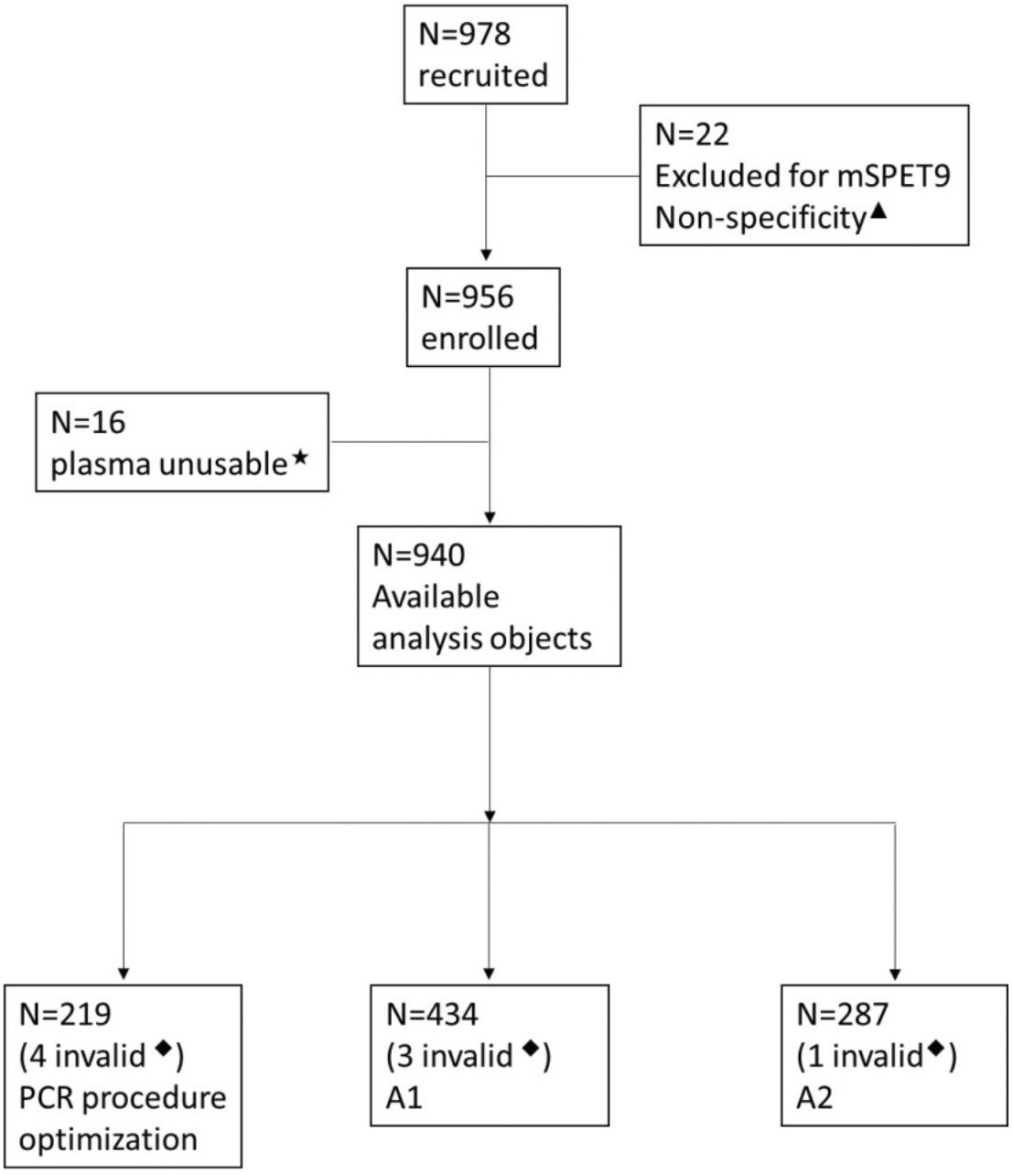
The distribution of all subjects. ▲, diagnosed with gastric cancer or hepatic carcinoma besides CRC; ★, insufficient plasma or cf‐DNA purification failed; ◆, invalid ACTB detection. A1, HotStart HiTaq DNA Polymerase used; A2, high affinity HotStart Taq used.

**FIGURE 2:**
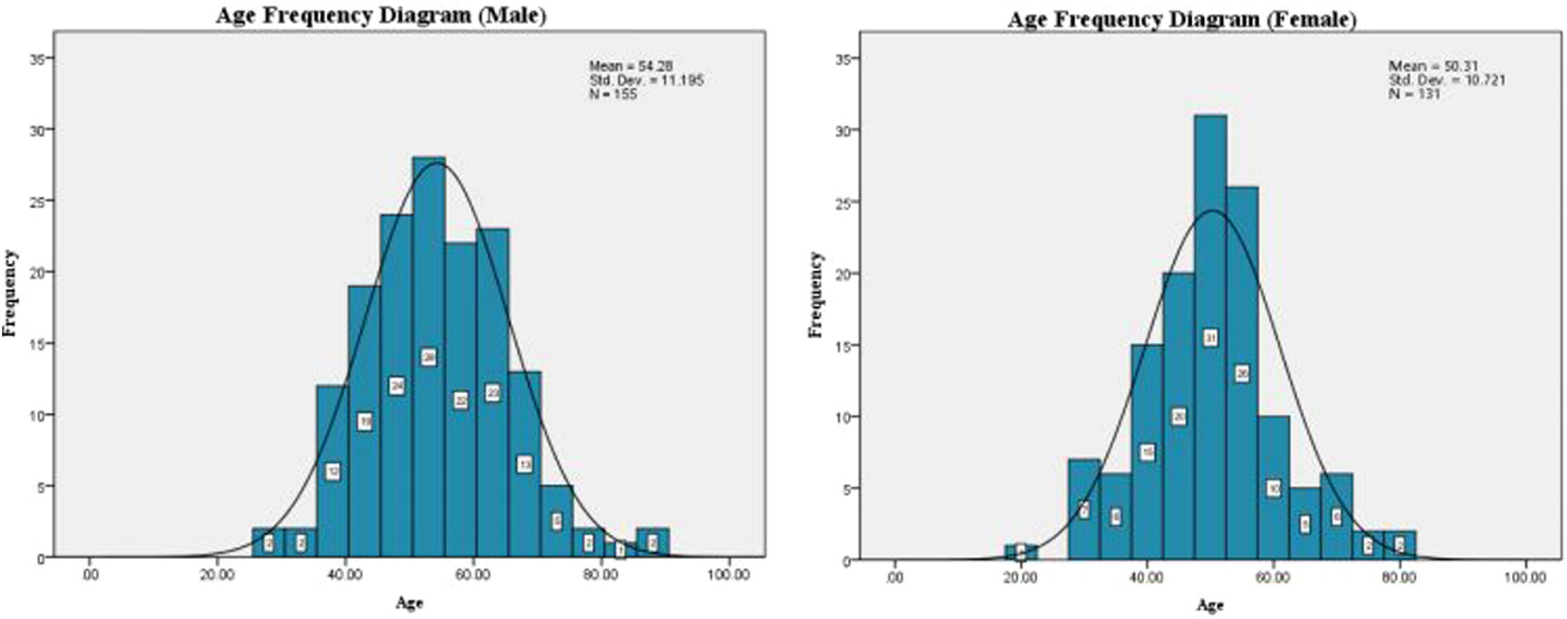
The age–gender distribution of valid subjects of A2.

Participants were recruited from October 2018 to June 2019 at five centers of mainland China, and 978 subjects were recruited. Twenty‐two subjects diagnosed with gastric cancer or hepatic carcinoma in addition to CRC were edged out, including 15 cases of gastric adenoma, 6 cases of gastrointestinal metaplasia, and 1 case of hepatic carcinoma, which might lead to a positive detection for mSEPT9.^45^, ^46^ Sixteen subjects were excluded for insufficient plasma or cf‐DNA purification failed. Eight subjects including 2 CRC, 3 NED, and 3PL were left out for their invalid ACTB detection. The 215 subjects including 62 CRC, 47 PL, and 106 NED were used during PCR procedure optimization. The 431 subjects including 137 CRC, 96 PL, and 198 NED were used for A1 assay as mentioned above. Ultimately, 286 subjects including 75 CRC, 40 PL, and 171 NED were used for A2 assay. Figure 1 gives the distribution of all subjects. Figure 2 gives the age–gender distribution, and Table 1 gives the characteristics of all valid subjects of A2. Tests of normality were executed, and both the male and female subjects were subject to standard normal distribution for their S‐W values were 0.267 and 0.193, respectively (Appendix S1). The youngest participant in the study was a 20‐year‐old young female volunteer, while the oldest an 88‐year‐old male CRC patient.

**TABLE 1:** The demographic characteristics of valid subjects of A2.

Group	Total	Gender		Age	
		Male (%)	Female (%)	Medium (range)	Mean
CRC	75	54 (72.0)	21 (28.0)	57 (28–88)	57
Right‐side disease^a^	19	15 (78.9)	4 (21.1)	55 (29–81)	57
Left‐side disease^a^	56	39 (69.6)	17 (30.4)	56.5 (28–88)	57
Stage I	7	5 (71.4)	2 (28.6)	58 (51–88)	64
Stage II	19	12 (63.2)	7 (36.8)	57 (40–68)	55
Stage III	18	16 (88.9)	2 (11.1)	57.5 (32–81)	59
Stage IV	3	1 (33.3)	2 (66.7)	49 (36–62)	49
Unknown stage	28	20 (71.4)	8 (28.6)	56.5 (28–82)	55.5
PL	40	27 (67.5)	13 (32.5)	50 (32–88)	53
NA	29	19 (65.5)	10 (34.5)	49 (32–69)	49
AA	11	8 (72.7)	3 (27.3)	69 (35–88)	62.4
NED	171	74 (43.3)	97 (56.7)	50 (20–78)	51
Total	286				

Abbreviations: AA, advanced adenoma; NA, non‐advanced adenoma; NED, no evidence of disease; PL, precancerous lesions.

^a^ Includes 1 case of descending colon sigmoid junction carcinoma and 3 cases of rectal sigmoid junction carcinoma.

### 3.2 Accuracy measurements

Table 2 gives the overall performance of the two assays. A2 shows superior detection performance compared to A1. Therefore, subsequent result analysis will focus on this assay.

**TABLE 2:** Performance of different PCR assays.

Assays	A1	A2
Sensitivity	83.9% (115/137)	82.7% (62/75)
Specificity	54.0% (107/198)	90.1% (154/171)
PL detection	57.3% (55/96)	55.0% (22/40)
Accuracy	66.3% (222/335)	87.8% (216/246)

### 3.3 Age and gender

**FIGURE 3:**
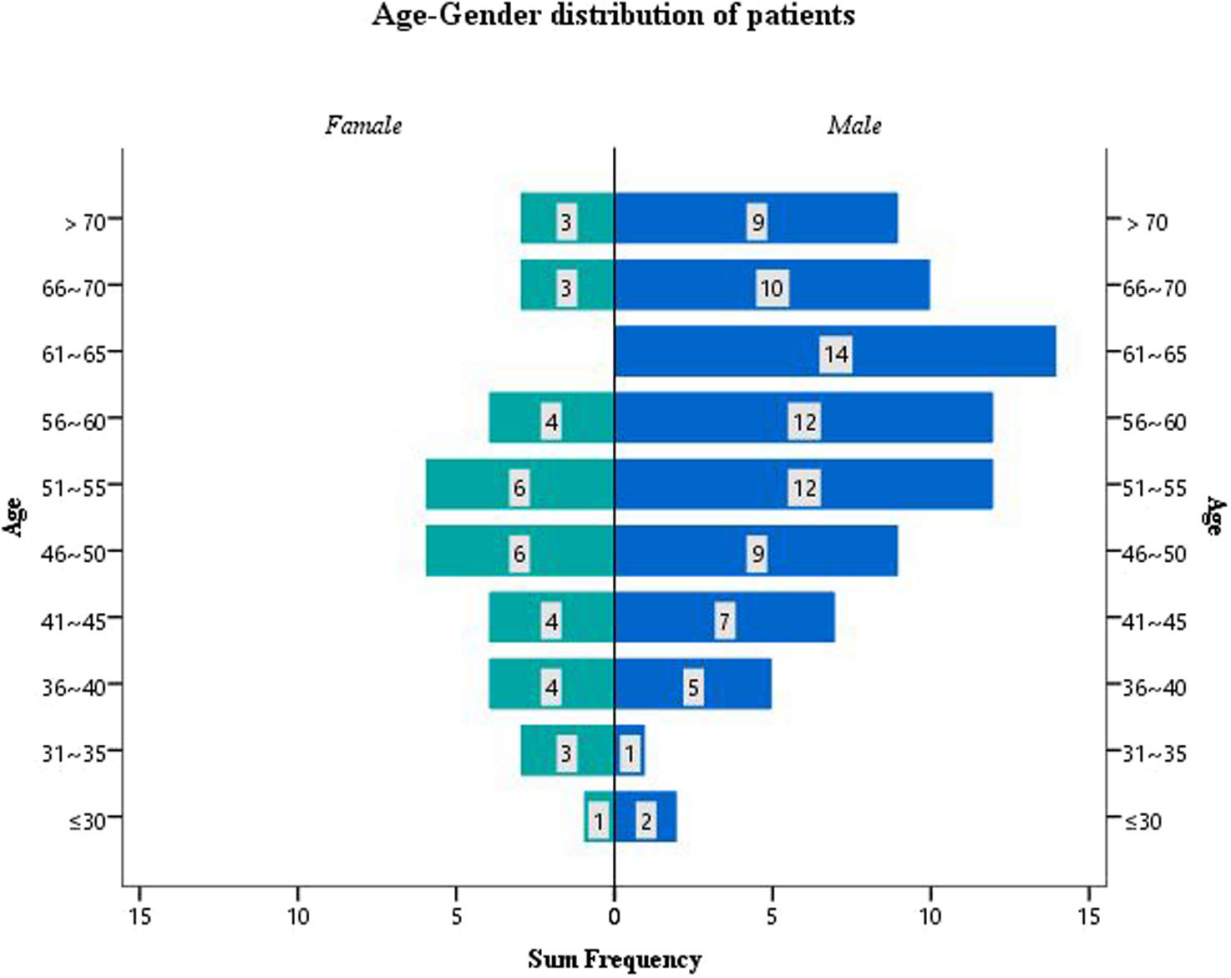
The age–gender distribution of PL and CRC subjects of A2.

Among the 115 PL or CRC patients, 67.8% were aged ≥50, and nearly 70% were male (Table 1). Men aged 61 ~ 65 bear the highest risk of CRC, and more than three‐quarters of the total number of male patients were aged ≥50. Figure 3 gives the age–gender distribution of patients. Table 3 demonstrates the positive detection rate of different categories of subjects which been tested. It turned out that this assay shared the similar performance in detecting CRC of males and females, standardized sensitivity values were 81.5% (95% CI 68.1–90.3) and 85.7% (95% CI 62.6–96.2), standardized specificity values were 91.2% (95% CI 82.6–96.7) and 88.7% (95% CI 80.2–93.9), respectively. There was a slight advantage in the detection of PL in people aged ≥50 years versus those <50 years and in women versus men.

**TABLE 3:** Positive detection rate of different categories of subjects tested by A2 assay.

Group	Pos./Tested (%)		
	PL	CRC	NED
Age			
<55	12/24 (50.0)	24/30 (80.0)	12/118 (10.2)
≥55	10/16 (62.5)	38/45 (84.4)	5/53 (9.4)
Gender			
Male	13/27 (48.2)	44/54 (81.5)	6/74 (8.1)
Female	9/13 (69.2)	18/21 (85.7)	11/97 (11.3)
Location			
Right‐side		17/19 (89.5)	
Left‐side		45/56 (80.4)	
Stage			
NA		14/29 (48.3)	
AA		8/11 (72.7)	
Stage I		5/7 (71.4)	
Stage II		14/19 (73.7)	
Stage III		15/18 (83.3)	
Stage IV		3/3 (100.0)	
Unknown stage		25/28 (89.3)	
Total stage		84/115 (73.0)	

Abbreviations: AA, advanced adenoma; CRC, colorectal cancer; NA, non‐advanced adenoma; PL, precancerous lesions.

### 3.4 Location and stages

Colorectal cancer detection rates were slightly underrepresented in the left side disease, mainly because the portion from the transverse colon to the rectum showed a different proportional decrease in its tumor detection capacity (Table 3). Moreover, the CRC detection showed a positive correlation with its progression, and the overall detection rate for all stages was 73.0% (95% CI 63.8–80.7).

### 3.5 Individual gene performance

**FIGURE 4:**
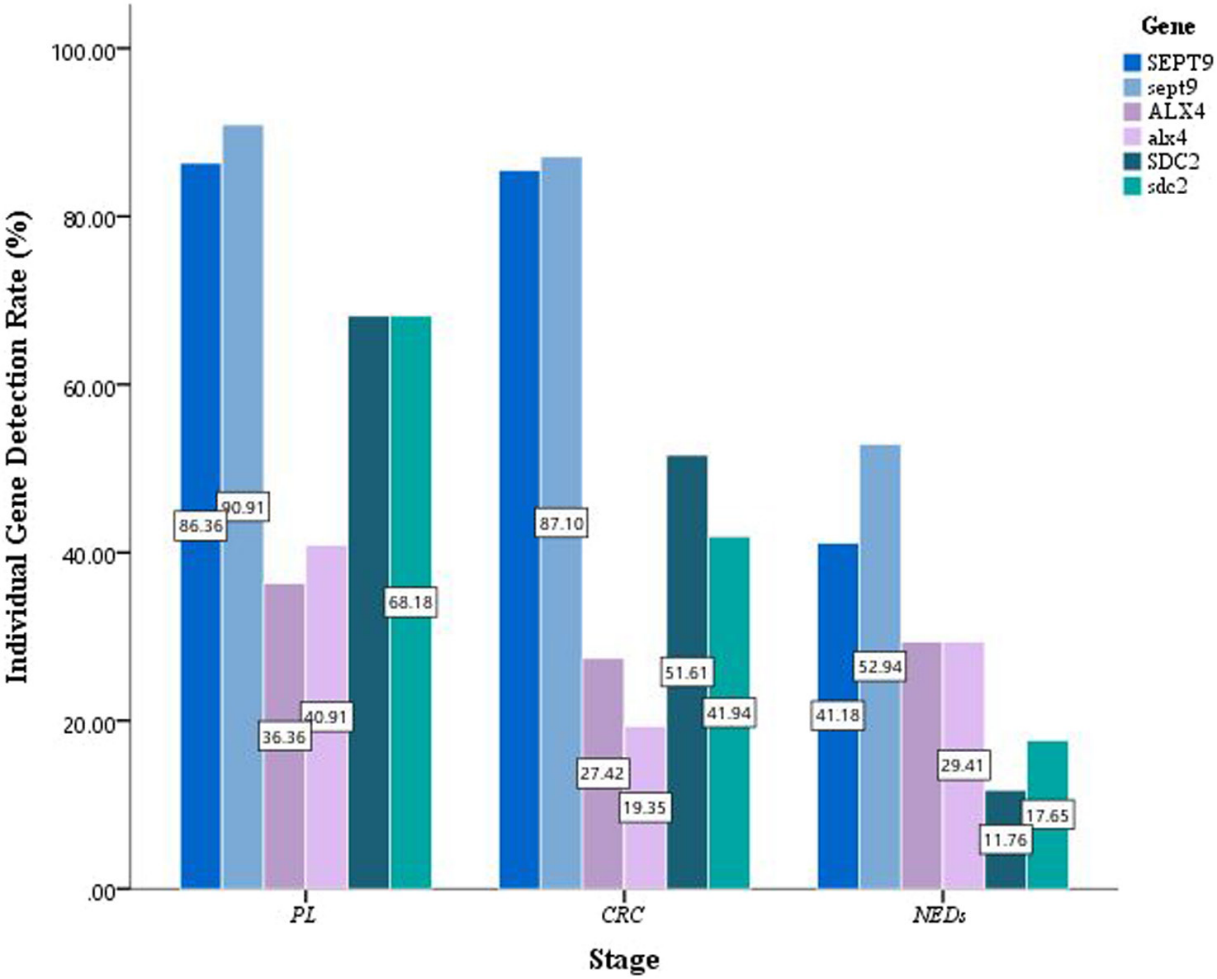
The individual gene detection rate by subject types. The upper and lower case of the gene name represent the detection rate of this methylated gene in two replicates, respectively. CRC, colorectal cancer; NED, no evidence of disease; PL, precancerous lesions.

For those samples that were judged to be positive, we analyzed their individual gene performance (Figure 4). If considered any of the conditions SEPT9 <45, ALX4 <38, and SDC2 <40 was met to be positive, individual genes performed roughly the same in two replicates. Nearly 85% of CRC or PL subjects, while over 40% of NEDs were detected for mSEPT9, implied that a higher false‐positive risk might be carried if the test results based on mSEPT9 alone. It was noteworthy that mSDC2 being even higher detected in PL than CRC and lowly in NEDs, which might be an ideal biomarker for early CRC detection. Compared with the former two, the performance of mALX4 was more moderate, and high or low detection of mALX4 was not found in all three types of samples.

### 3.6 mALX4 effect

mALX4 seems to perform almost uniformly across all sample types and has no significant impact on the results. We removed mALX4 and reanalyzed the result to compare with the existence of mALX4 in A2 assay. It showed that the presence of mALX4 had a tiny effect on the overall accuracy but definitely improved the sensitivity and detection of PL at the expense of some specificity. Table 4 shows the effect of the presence or absence of the mALX4 on the results of A2 assay.

**TABLE 4:** Effect of mALX4 of A2 assay.

A2 assay	mALX4 presence	mALX4 absence
Sensitivity	82.7% (62/75)	74.7% (56/75)
Specificity	90.1% (154/171)	95.3% (163/171)
PL detection	55.0% (22/40)	50.0% (20/40)
Accuracy	87.8% (216/246)	89.0% (219/246)

Abbreviation: PL, precancerous lesions.

### 3.7 Duplicates coincide

The consistency of the results between the two replicates of A2 assay was moderate intensity, and 73.1% (209/286) of subjects exhibited two consistent results (*κ* = 0.450, 95% CI 0.440–0.460, *p* < 0.001). For details, please refer to Appendix S1.

## 4 DISCUSSION

Cancer patients have higher levels of cell‐free DNA in their plasma, which likely comes from tumor cells.^47^, ^48^ Abnormal DNA methylation is closely linked to tumor development, including colorectal cancer, and can disrupt gene function.^10^, ^11^, ^49^, ^50^, ^51^ Cell‐free DNA methylation in plasma is higher in cancer patients compared to healthy individuals,^12^, ^52^ and different molecular markers show significant differences across the stages of colorectal cancer.^53^ Detection of cell‐free DNA methylation in plasma is thus an important tool for early screening of colorectal cancer.^13^, ^15^, ^25^, ^54^, ^55^ Abnormal methylation of SEPT9 gene is the most widely used molecular marker of cell‐free DNA in CRC screening. Unfortunately, the sensitivity of methylated SEPT9 in detecting advanced adenomas is only around 9.8%–21.6%, indicating that relying solely on methylated SEPT9 as a screening strategy for colorectal precancerous lesions is insufficient.^56^, ^57^

In the present study, we compared our new multitarget plasma DNA assay (named ColoProbe) with colonoscopy among colorectal cancer patients and healthy persons. ColoProbe is a combined assay that can simultaneously detect three methylation biomarkers, namely SEPT9, SDC2, and ALX4, in a single qPCR reaction. Through the ColoProbe test, we observed significant methylation of all three genes (SEPT9, SDC2, and ALX4) in CRC tissues compared to paired paracancerous tissues (*p* < 0.01). ColoProbe test showed 82.7% sensitivity for detecting CRC and 55.0% for PL detection with a specificity of 90.1% in distinguishing CRC and PL subjects from normal subjects. When compared with Epi proColon 2.0, ColoProbe test has a huge advantage in detecting precancerous lesions (Appendix S1), likely because the detection of three markers promoted the LoD of the assay (Table 2). The improvement in detection of precancerous lesions is of particular importance, since it provides an opportunity to stop the lesions from progressing into invasive diseases. Therefore, a high‐quality plasma screening test evaluation criterion should include both PL and CRC detection performance.

In our previous study, we have identified mTFPI2 (tissue factor pathway inhibitor 2) as a potential molecular marker for CRC screening.^58^, ^59^, ^60^, ^61^, ^62^, ^63^, ^64^ Further study revealed that mTFPI2 was elevated in gastric cancer and hepatocellular carcinoma,^63^, ^65^, ^66^, ^67^ even in inflamed colon tissue,^68^ mTFPI2 should be more than just a CRC biomarker.^69^, ^70^, ^71^, ^72^ A similar situation was observed with mSEPT9, where SEPT9 gene methylation was detected in a significant percentage of gastric cancer, head and neck squamous carcinoma, hepatic carcinoma, esophageal cancer, lung cancer, cholangiocarcinoma, and bladder cancer patients.^45^, ^73^, ^74^, ^75^, ^76^ As cancer development is influenced by multiple factors and genes,^77^ it would be unfair to assess the risk of a particular cancer solely based on the results of a single gene methylation test. Detection of multiple markers may yield a more comprehensive screening effect, covering various molecular pathways involved in tumor formation and enhancing detection sensitivity. Compared with single gene methylation detection, studies also have found that the combination of more genes can cover multiple molecular pathways of tumor formation and further improve detection sensitivity.^78^

The SDC2 gene, known for its high methylation level in CRC tumor tissues compared to adjacent non‐tumor tissues, has demonstrated good detection performance as a mature methylated marker for CRC.^28^ SDC2 is expressed in colon mesenchymal cells and is involved in cell division and migration.^30^, ^79^ Several studies have reported high sensitivity and specificity of methylated SDC2 for CRC screening in serum, plasma, or stool samples.^29^, ^30^ Recently, stool methylated SDC2 tests for CRC screening have also been reported, showing sensitivities ranging from 81.1% to 90.0% for detecting CRC and 33.3% to 58.2% for detecting advanced adenomas, with specificities of 90.9%–93.3%.^30^, ^80^ In our study, plasma methylated SDC2 test showed a lower sensitivity for detecting CRC. Such a difference could be due to lower cell‐free DNA level in plasma than tumor DNA in stool. On the contrary, SDC2 has a low false positive rate in NEDs, which was quite comparable to those of Cologuard^81^ and stool SDC2 tests,^30^, ^80^ thus plays a great role in improving the specificity of early screening.

Salehi et al.^31^ reported a sensitivity of 68% and specificity of 88% were achieved in the detection of promoter methylation of ALX4 in colorectal neoplasia samples. Our results showed that the sensitivity of ALX4 gene in CRC and PL alone was inferior to the other two markers, SEPT9 and SDC2, besides ALX4 had a higher false‐positive rate in NEDs. The sensitivity of ColoProbe to detect CRC is 81.6%, the specificity of PL is 58.5%, and the specificity is 91.0%. And we found that the sensitivity of three‐gene combined detection was significantly higher than that of two‐gene detection. Therefore, combined detection of ALX4 can further improve the sensitivity of CRC and PL while still maintaining a high level of specificity.

Considering that the cost of the kit primarily consists of the primers and probes, we conducted a cost comparison between the testing of the mSEPT9 gene alone and the simultaneous testing of the three genes' methylation. Remarkably, we found that the combined testing of the three genes resulted in a mere 2.72% increase in cost. This indicates that, in addition to its broader coverage of molecular pathways associated with tumor formation, the multi‐gene approach has minimal impact on the overall cost of detection. Thus, it remains a viable and efficient method for early screening compared to the detection of single gene methylation.

### 4.1 Limitations

Some implausibility exists in this study. First, the NEDs in this study were mainly from the north of China, while the patients were mainly from the south of China, the population may not be typical. Secondly, the performance of ALX4 in this study was unsatisfactory, and it needs to be more for further study. Thirdly, we did not follow up the patients who tested positive. The NEDs in the test may not be absolutely healthy because these persons have not been checked by the gold standard. Finally, the proportion of circulating tumor DNA (ctDNA) to total cell‐free DNA (cfDNA) is low, and the relevant marker loci may not be detected by a single test. The need for repeated testing greatly increases the testing cost and testing cycle.

## 5 CONCLUSION

In summary, the combined detection of SEPT9, SDC2, and ALX4 methylation status in plasma can cover multiple molecular pathways of tumor formation and further improvement in detection sensitivity, especially of the PL. ColoProbe assay is expected to become a new, noninvasive method for screening colorectal cancer. Further validation is warranted.

## AUTHOR CONTRIBUTIONS

**Yuan Li:** Conceptualization (equal); formal analysis (equal); writing – original draft (equal). **Bin Li:** Conceptualization (equal); formal analysis (equal); writing – original draft (equal). **Rou Jiang:** Data curation (equal); formal analysis (equal); writing – original draft (equal). **Leen Liao:** Data curation (equal). **Chunting Zheng:** Data curation (equal). **Jie Yuan:** Data curation (equal). **Liuhong Zeng:** Data curation (equal). **Kunling Hu:** Data curation (equal). **Yuyu Zhang:** Methodology (equal). **Weijian Mei:** Methodology (equal). **Zhigang Hong:** Methodology (equal). **Binyi Xiao:** Methodology (equal). **Lingheng Kong:** Methodology (equal). **Kai Han:** Methodology (equal). **Jinghua Tang:** Methodology (equal). **Wu Jiang:** Methodology (equal). **Zhizhong Pan:** Supervision (equal); writing – review and editing (equal). **Shenyan Zhang:** Supervision (equal); writing – review and editing (equal). **Peirong Ding:** Conceptualization (equal); supervision (equal); writing – review and editing (equal).

## FUNDING INFORMATION

The study was supported by the National Natural Science Foundation of China (grant numbers 81871971, 82073159) and the Sun Yat‐sen University Clinical Research 5010 Program (grant number 2014013).

## CONFLICT OF INTEREST STATEMENT

We declare that we have no financial and personal relationships with other people or organizations that can inappropriately influence our work, there is no professional or other personal interest of any nature or kind in any product, service, and/or company that could be construed as influencing the position presented in, or the review of, the manuscript entitled.

## CONSENT FOR PUBLICATION

Written informed consent for publication was obtained from all participants.

## ETHICS STATEMENT

All procedures performed in studies involving human participants were in accordance with the ethical standards of the institutional and/or national research committee and with the 1964 Helsinki Declaration and its later amendments or comparable ethical standards. The study was approved by the Institutional Research Ethics Committee of Sun Yat‐sen University Cancer Center (approval number: B2018‐105‐01).

## Supporting information


Appendix S1

## Data Availability

The authenticity of this article has been validated by uploading the key raw data onto the Research Data Deposit public platform (www.researchdata.org.cn).
