# Supplementary material for: A novel screening method of DNA methylation biomarkers helps to improve the detection of colorectal cancer and precancerous lesions
Source: Cancer Med. 2023 Oct 25;12(21):20626–38. doi: 10.1002/cam4.6511 (PMC10660402; doi:10.1002/cam4.6511)
Supplement: Supplementary file 1 — Appendix S1 [file CAM4-12-20626-s001.docx]

**Datas Supplementary**

**Table S1** Tests of normality of A2 subjects. A. female; B.male

**A**

| **Case Processing Summary** | | | | | | |
| --- | --- | --- | --- | --- | --- | --- |
|  | Cases | | | | | |
|  | Valid | | Missing | | Total | |
|  | N | Percent | N | Percent | N | Percent |
| female | 131 | 84.5% | 24 | 15.5% | 155 | 100.0% |

| **Descriptives** | | | | |
| --- | --- | --- | --- | --- |
|  | | | Statistic | Std. Error |
| female | Mean | | 50.305 | .936 |
|  | 95% Confidence Interval for Mean | Lower Bound | 48.452 |  |
|  |  | Upper Bound | 52.158 |  |
|  | 5% Trimmed Mean | | 50.117 |  |
|  | Median | | 50.000 |  |
|  | Variance | | 114.937 |  |
|  | Std. Deviation | | 10.721 |  |
|  | Minimum | | 20.000 |  |
|  | Maximum | | 82.000 |  |
|  | Range | | 62.000 |  |
|  | Interquartile Range | | 11.000 |  |
|  | Skewness | | .263 | .212 |
|  | Kurtosis | | .631 | .420 |

| **Tests of Normality** | | | | | | |
| --- | --- | --- | --- | --- | --- | --- |
|  | Kolmogorov-Smirnov^a^ | | | Shapiro-Wilk | | |
|  | Statistic | df | Sig. | Statistic | df | Sig. |
| female | .086 | 131 | .018 | .986 | 131 | .193 |

a. Lilliefors Significance Correction

**B．**

| **Case Processing Summary** | | | | | | |
| --- | --- | --- | --- | --- | --- | --- |
|  | Cases | | | | | |
|  | Valid | | Missing | | Total | |
|  | N | Percent | N | Percent | N | Percent |
| male | 155 | 100.0% | 0 | 0.0% | 155 | 100.0% |

| **Descriptives** | | | | |
| --- | --- | --- | --- | --- |
|  | | | Statistic | Std. Error |
| male | Mean | | 54.277 | .899 |
|  | 95% Confidence Interval for Mean | Lower Bound | 52.501 |  |
|  |  | Upper Bound | 56.054 |  |
|  | 5% Trimmed Mean | | 54.043 |  |
|  | Median | | 53.000 |  |
|  | Variance | | 125.319 |  |
|  | Std. Deviation | | 11.195 |  |
|  | Minimum | | 28.000 |  |
|  | Maximum | | 88.000 |  |
|  | Range | | 60.000 |  |
|  | Interquartile Range | | 17.000 |  |
|  | Skewness | | .308 | .195 |
|  | Kurtosis | | .221 | .387 |

| **Tests of Normality** | | | | | | |
| --- | --- | --- | --- | --- | --- | --- |
|  | Kolmogorov-Smirnov^a^ | | | Shapiro-Wilk | | |
|  | Statistic | df | Sig. | Statistic | df | Sig. |
| male | .054 | 155 | .200^*^ | .989 | 155 | .267 |

*. This is a lower bound of the true significance.

a. Lilliefors Significance Correction


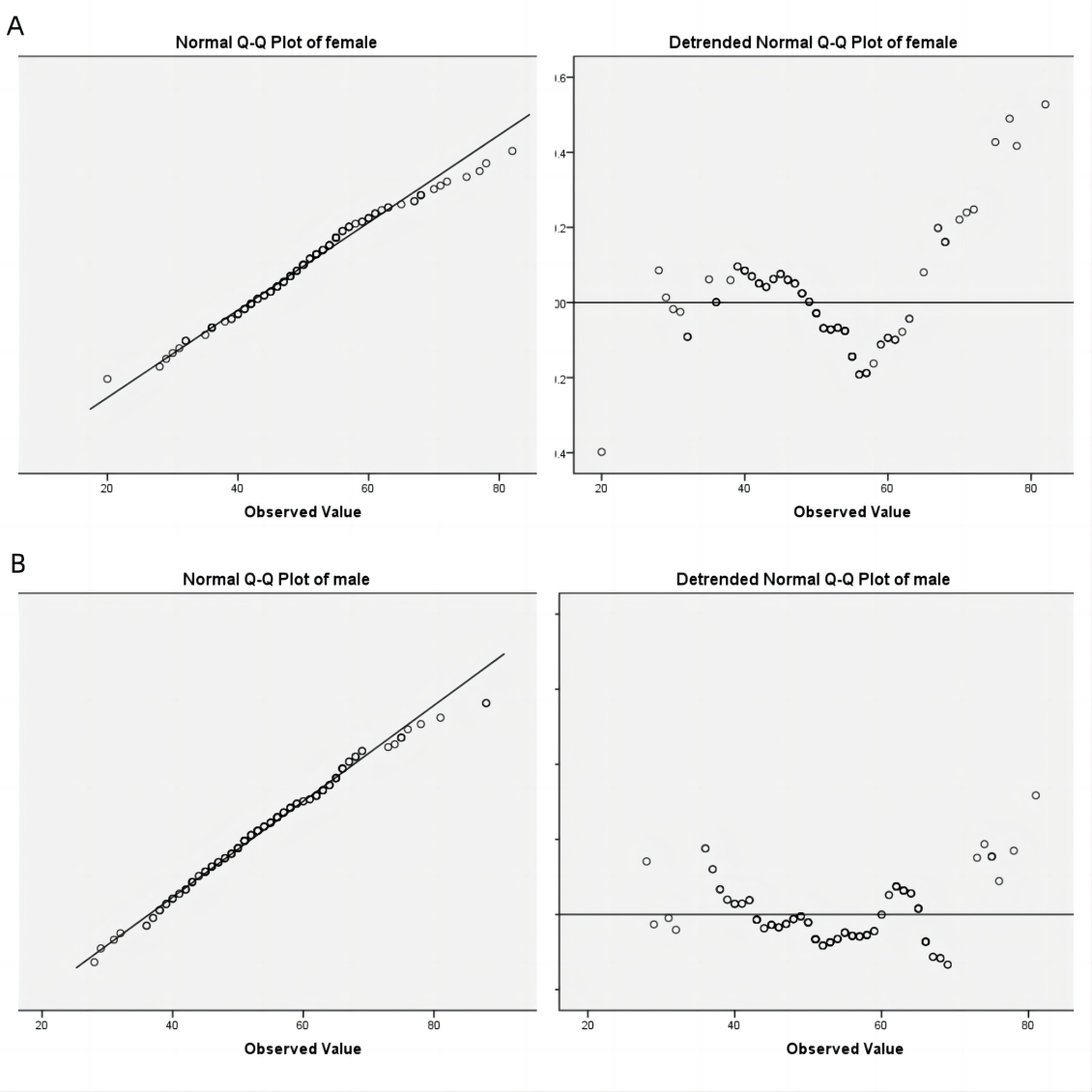


**Fig S1.** Tests of normality of A2 subjects. A. female; B.male

**Table S2.** The consistency of the results between the two replicates of A2 assay.

| **Case Processing Summary** | | | | | | |
| --- | --- | --- | --- | --- | --- | --- |
|  | Cases | | | | | |
|  | Valid | | Missing | | Total | |
|  | N | Percent | N | Percent | N | Percent |
| Dia1 * Dia2 | 286 | 100.0% | 0 | 0.0% | 286 | 100.0% |

| **Dia1 * Dia2 Crosstabulation** | | | | | |
| --- | --- | --- | --- | --- | --- |
|  | | | Dia2 | | Total |
|  |  |  | Negative | Positive |  |
| Dia1 | Negative | Count | 125.0 | 36.0 | 161.0 |
|  |  | Expected Count | 93.4 | 67.6 | 161.0 |
|  |  | % within Dia1 | 77.6% | 22.4% | 100.0% |
|  |  | % within Dia2 | 75.3% | 30.0% | 56.3% |
|  |  | % of Total | 43.7% | 12.6% | 56.3% |
|  | Positive | Count | 41.0 | 84.0 | 125.0 |
|  |  | Expected Count | 72.6 | 52.4 | 125.0 |
|  |  | % within Dia1 | 32.8% | 67.2% | 100.0% |
|  |  | % within Dia2 | 24.7% | 70.0% | 43.7% |
|  |  | % of Total | 14.3% | 29.4% | 43.7% |
| Total | | Count | 166.0 | 120.0 | 286.0 |
|  |  | Expected Count | 166.0 | 120.0 | 286.0 |
|  |  | % within Dia1 | 58.0% | 42.0% | 100.0% |
|  |  | % within Dia2 | 100.0% | 100.0% | 100.0% |
|  |  | % of Total | 58.0% | 42.0% | 100.0% |

| **Symmetric Measures** | | | | | |
| --- | --- | --- | --- | --- | --- |
|  | | Value | Asymp. Std. Error^a^ | Approx. T^b^ | Approx. Sig. |
| Measure of Agreement | Kappa | .450 | .053 | 7.622 | .000 |
| N of Valid Cases | | 286 |  |  |  |

1. Not assuming the null hypothesis.

b. Using the asymptotic standard error assuming the null hypothesis.

**Table S3.** Comparison of the detection of precancerous lesions between Epi proColon and ColoProbe.

|  | NA | AA | StageⅠ | StageⅡ | StageⅢ | StageⅣ |
| --- | --- | --- | --- | --- | --- | --- |
| ColoProbe | 48.3%（14/29） | 72.7%（8/11） | 71.4%（5/7） | 73.7%（14/19） | 83.3%（15/18） | 100%（3/3） |
| Epi proColon^a^ | 7.7%（16/209） | 9.6%（30/314） | 36.4%（8/22） | 57.1%（8/14） | 58.3%（7/12） | 80.0%（4/5） |
| Epi proColon 2.0^b^ |  | 27.4% | 66.7% | 82.6% | 84.1% | 100% |

1. Church, T.R., et al., Prospective evaluation of methylated SEPT9 in plasma for detection of asymptomatic colorectal cancer. Gut, 2014. 63(2): p. 317-25.
2. Jin, P., et al., Performance of a second-generation methylated SEPT9 test in detecting colorectal neoplasm. J Gastroenterol Hepatol, 2015. 30(5): p. 830-3.

**Methods Supplementary**

**A.**

**
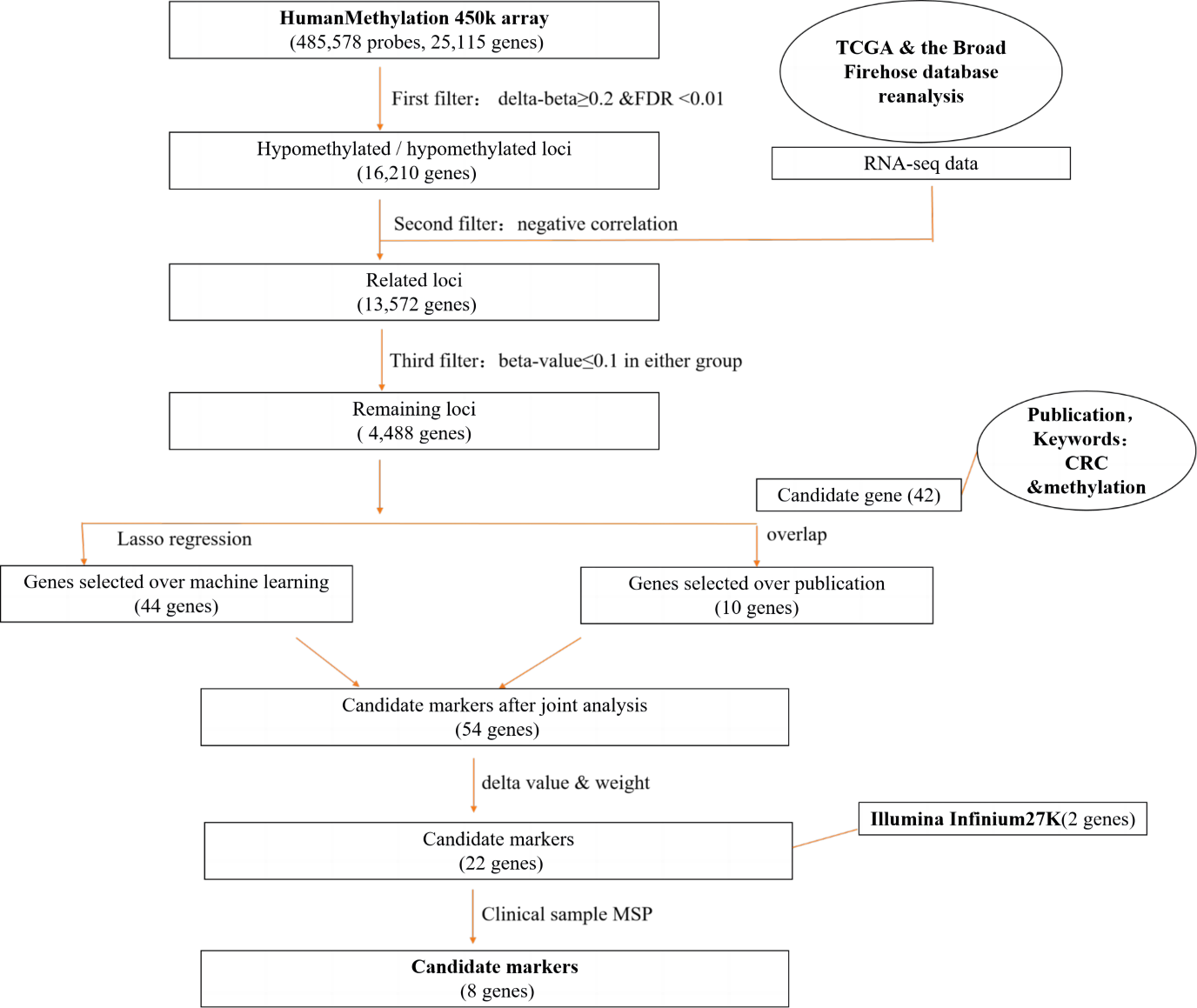
**

**B.**

**
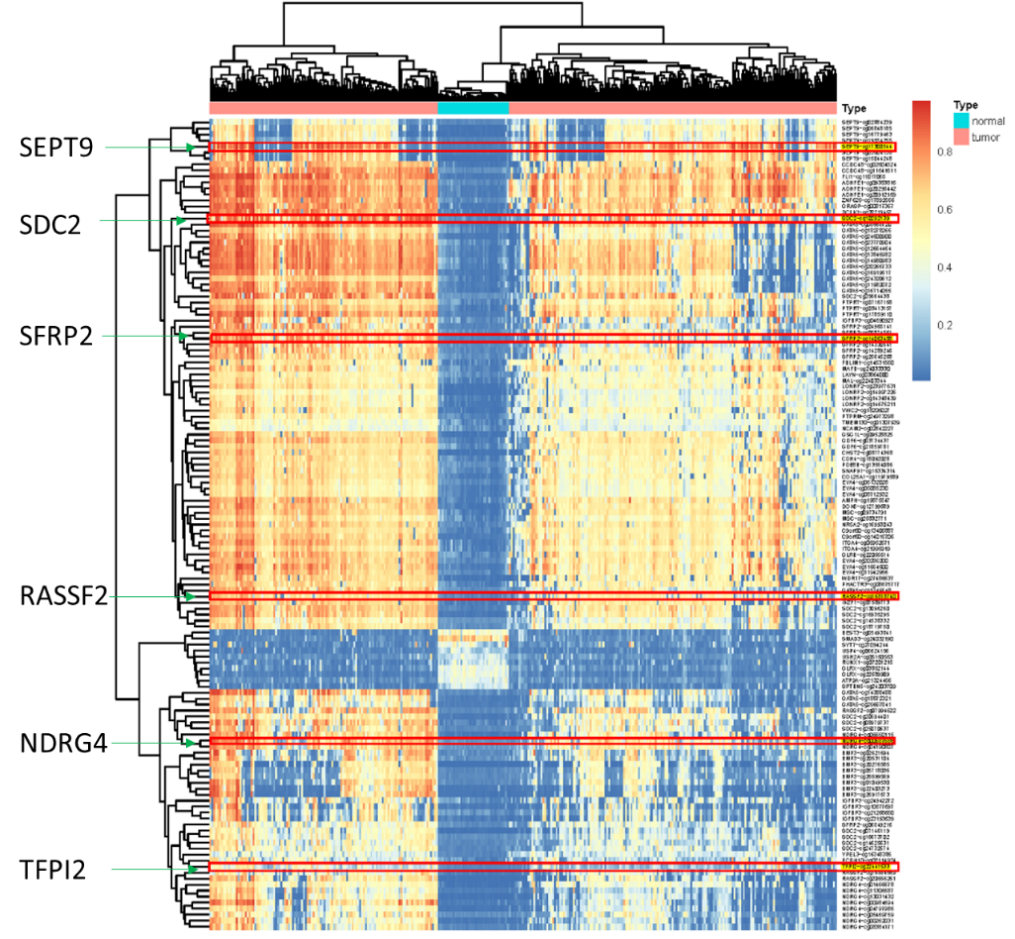
**

**C.**

**
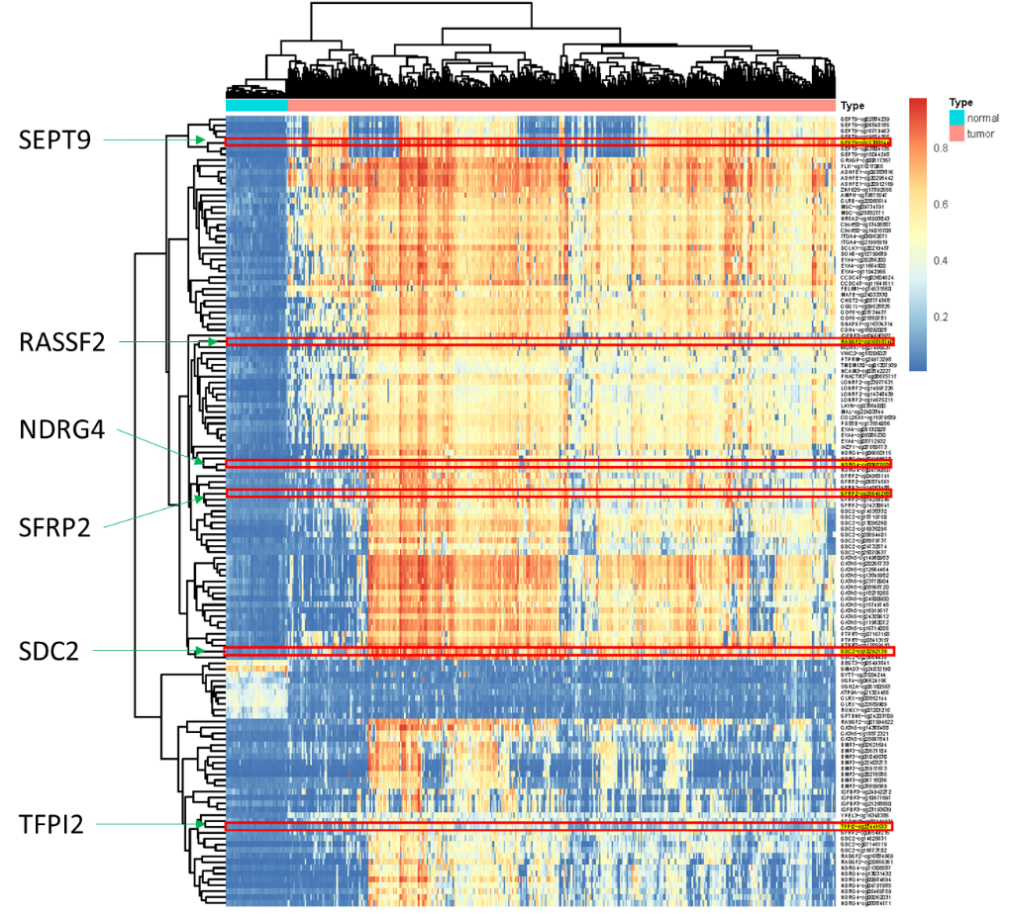
**

**Fig S1. CRC biomarkers selection and confirmation.** A. Schematic of the biomarker selection process; B. Cluster analysis on chip data of 297 colon adenocarcinoma samples and 38 matched adjacent normal tissue samples (vertical, annotation genes, horizontal, samples); C. Cluster analysis on chip data of 396 colorectal adenocarcinoma samples and 35 matched adjacent normal tissue samples (vertical, annotation genes, horizontal, samples)

**Table S1. Performance of the 8 selected CRC biomarkers.**

| **Biomarkers** | **SEPT9** | **SDC2** | **TFPI2** | **ALX4** | **SFRP2** | **RASSF2** | **NDRG4** | **CHFR** |
| --- | --- | --- | --- | --- | --- | --- | --- | --- |
| Sensitivity for tissues | 100%,  6/6 | 83.3%,  5/6 | 83.3%,  5/6 | 100%,  6/6 | 83.3%,  5/6 | 85.7%,  6/7 | 71.4%,  5/7 | 57.1%, 4/7 |
| Specificity for plasma | 85.0%,  68/80 | 98.3%,  59/60 | 98.3%,  59/60 | 97.5%,  78/80 | 96.7%,  58/60 | 95.0%,  57/60 | 91.7%,  55/60 | 82.5%, 33/40 |

**A.**


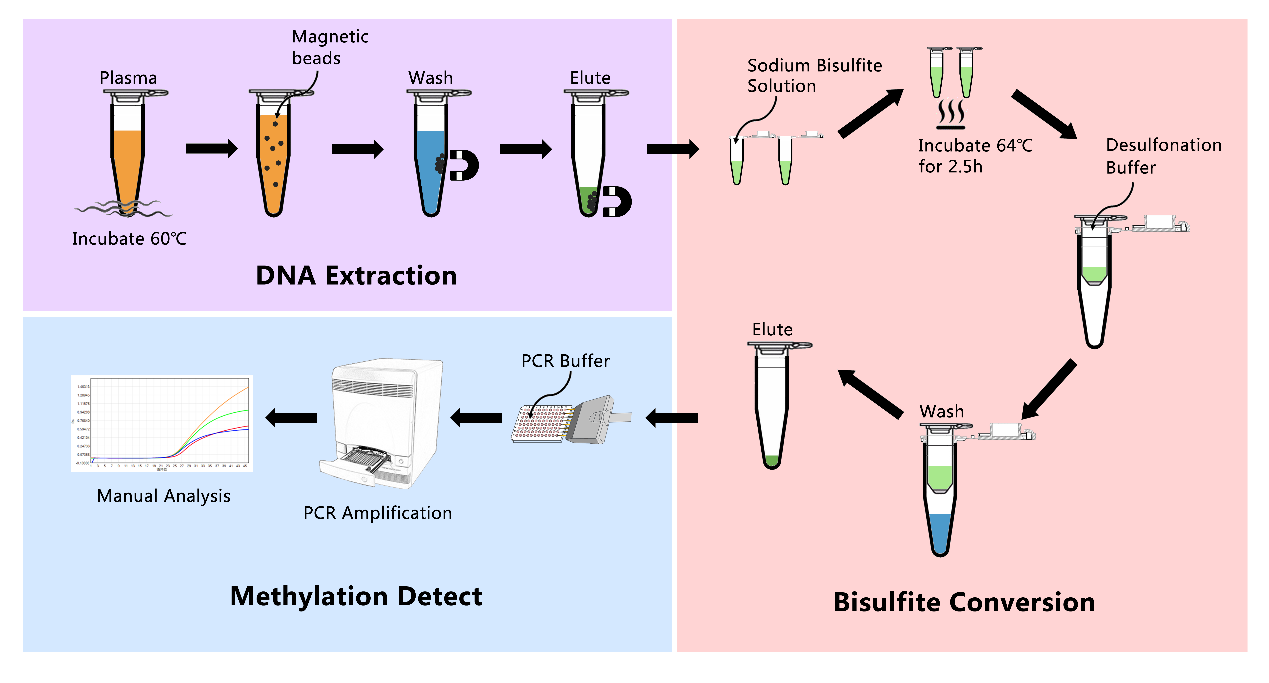


**B.**

| Components | Volume(ul) | Final concentration |
| --- | --- | --- |
| High Affinity HotStart Taq | 0.25 |  |
| 5×Probe qPCR Buffer | 5 | 1× |
| 10×HA Buffer | 2.5 | 1× |
| dNTPs（25mM） | 0.2 | 200μM |
| ACTB Primer F（10μM） | 0.75 | 300nM |
| ACTB Primer R（10μM） | 0.75 | 300nM |
| AXL4 Primer F（10μM） | 0.75 | 300nM |
| AXL4 Primer R（10μM） | 0.75 | 300nM |
| SDC2 Primer F（10μM） | 0.75 | 300nM |
| SDC2 Primer R（10μM） | 0.75 | 300nM |
| SEPT9 Primer F（10μM） | 0.75 | 300nM |
| SEPT9 Primer R（10μM） | 0.75 | 300nM |
| ACTB Probe（10μM） | 0.5 | 200nM |
| AXL4 Probe（10μM） | 0.5 | 200nM |
| SDC2 Probe（10μM） | 0.5 | 200nM |
| SEPT9 Probe（10μM） | 0.5 | 200nM |
| Nucleases Free Water | 4.05 |  |
| Mixture | 20 |  |

**C.**

| Procedure | | | | | | | |
| --- | --- | --- | --- | --- | --- | --- | --- |
| Initial step | PhasesⅠ （5 Cycles）^✦^ | | | PhasesⅡ（45 Cycles）^✧^ | | | |
| Polymerase activation | Denaturation | Annealing | Extension | Denaturation | Annealing 1 | Annealing 2 | Extension |
| 95℃，10min | 94℃，30s | Starting at 64.5°C, decrease 0.9°C per cycle until 60.9°C for 45s per cycle | 72℃，45s | 94℃, 30s | 60℃, 12s | 51℃, 33s | 70℃，45s  Plate Read |

✦ PhasesⅠ：a touchdown PCR procedure, an annealing step between degeneration and extension is excluded, which is starting at 64.5°C, decrease 0.9°C per cycle until 60.9°C for 45s per cycle, executing 5 cycles;

✧ PhasesⅡ：a conventional PCR procedure，which including the well-known denaturation- annealing- extension steps, the step annealing 1, probes annealing; step annealing 2, primers annealing.

**D.**

| **Oligos** | **Sequence** | **Purification** | **5’ modification** | **3’modification** |
| --- | --- | --- | --- | --- |
| ACTB Primer F | 5′-TGGTGATGGAGGAGGTTTAGTAAGT | PAG |  |  |
| ACTB Primer R | 5′-AACCAATAAAACCTACTCCTCCCTTAA | PAG |  |  |
| AXL4 Primer F | 5’-TGATATTTTAGTTAGGGTATTTGCG | PAG |  |  |
| AXL4 Primer R | 5’-TCAAAACTTAATAACTCCGAC | PAG |  |  |
| SDC2 Primer F | 5’-GTACGGGAAAGGAGTTCGC | PAG |  |  |
| SDC2 Primer R | 5’-ATTTCTACACTCCCGACACGA | PAG |  |  |
| SEPT9 Primer F | 5'-GTTGTTTCGGAATTTTTTAGGAC | PAG |  |  |
| SEPT9 Primer R | 5'-CAAAATCCTCTCCAACACGTC | PAG |  |  |
| ACTB Probe | 5-ACCACCACCCAACACACAATAACAAACACA | HPL | FAM | BHQ1 |
| AXL4 Probe | 5-GATTTTAATGCGAAGTTTTAAGCGGT | HPL | ROX | BHQ2 |
| SDC2 Probe | 5-GTAAAATTATAGTAGAGTAAGAAGAGT | HPL | CY5 | BHQ3 |
| SEPT9 Probe | 5-GTTTTATTTTGGGAAATTTATCGGT | HPL | VIC | BHQ1 |

**Fig and Table S2. Technical details of ColonProbe detection.** A. Flowchart of ColonProbe, cf-DNA is purified by MagPure Circulating DNA Maxi Kit and bisulfited by EZ DNA Methylation-Gold Kit and then detected by a 7500 Real-Time PCR system; B. Mixture components; C. PCR assay process settings; D. Oligonucleotide sequences
